# Correction of Anophthalmic Enophthalmos with a Three-Staged Procedure: Two Case Reports

**DOI:** 10.1155/2011/731259

**Published:** 2011-09-21

**Authors:** Tomohiro Minagawa, Ryuta Shioya, Takeshi Yamao, Chigusa Sato, Taku Maeda

**Affiliations:** ^1^Department of Plastic and Reconstructive Surgery, Asahikawa Kosei General Hospital, 1-jo-dohri 24-chome 111, Asahikawa, Hokkaido 078-8211, Japan; ^2^Department of Plastic and Reconstructive Surgery, Hokkaido University Graduate School of Medicine, Sapporo, Hokkaido 060-0815, Japan

## Abstract

Surgical correction of an anophthalmic enophthalmos secondary to inappropriate repair of the eye socket involves several difficult aesthetic issues associated with long-term use of a poorly fitting prosthetic eye. In this paper, we present two cases of anophthalmic enophthalmos. During the treatment of the first patient, unsatisfactory cosmetic problems including lower eyelid retraction, hypoglobus, and severe upper eyelid ptosis were revealed. Accordingly, a three-staged procedure was performed on the second patient, including autologous augmentation of the eye socket, correction of lower eyelid retraction with a cartilage graft, and a frontalis sling procedure to correct upper eyelid ptosis.

## 1. Introduction

Enophthalmos due to inappropriate primary repair of the eye socket is a serious cosmetic complaint of patients with anophthalmia. The goal of surgical correction of anophthalmic enophthalmos is to provide orbital symmetry relative to the unaffected side; however, there are several cosmetic concerns, including lower eyelid retraction, hypoglobus, and sever upper eyelid ptosis. Here we report a case in which a patient experienced an unfavorable cosmetic outcome during restoration of orbital asymmetry. Furthermore, we present the result of a three-staged procedure performed systematically on a patient requiring correction of enophthalmos.

## 2. Case Presentation

### 2.1. Case  1

The first patient was a 54-year-old woman who underwent enucleation of the left eyeball after a motor vehicle accident three years before her visit to our hospital. She presented with a sunken eye and ptotic upper eyelid (Figure 1). We first performed augmentation of the eye socket using costal cartilage grafts under general anesthesia to improve her enophthalmos. Eight months postoperatively, levator aponeurosis repair was performed under local anesthesia to correct the ipsilateral upper eyelid ptosis. After the second surgery, the position of the corrected upper eyelid was balanced with the unaffected side, but appearance was unnatural (Figure 2). However, during the first few months after aponeurotic surgery, upper eyelid ptosis recurred (Figure 3). The cosmetic defects were likely due to the lower eyelid retraction and downgazing of prosthetic eye. Moreover, the recurrence of the upper eyelid ptosis may be associated with mechanical stress arising from insertion and removal of the prosthetic eye, which was heavier, thicker, and irregular (Figures 4(a) and 4(b)).

### 2.2. Case  2

The second patient was a 60-year-old woman with a recurrent schwannoma arising from the middle cranial base, who had undergone orbital exenteration at the age of 46. She complained of a sunken eye and drooping of the affected upper eyelid (Figure 5). Given the unfavorable findings of the first patient, we planned the following three-staged procedure: augmentation of the eye socket, correction of lower eyelid retraction and long-lasting correction of upper eyelid ptosis. First, augmentation of the eye socket was performed under general anesthesia. As a large portion of her orbital tissue had already been removed, free flap transfer was considered more preferable for orbital augmentation [1]; the patient refused to undergo this invasive surgery. Instead, we used an autologous ball-shaped graft composed of diced costal cartilage wrapped with the serratus fascia into the eye socket (Figures 6(a) and 6(b)) [2]. Four months later, the second- and third-stage procedures were performed under general anesthesia. We used an auricular cartilage graft for lower eyelid retraction, and a fascia lata graft for the frontalis sling to correct upper eyelid ptosis (Figure 7). The patient had an uneventful postoperative clinical course after the first and second operations. During 17 months of followup, the patient has been satisfied with the aesthetic improvement of the affected eye, although her enophthalmos was partially corrected (Figure 8).

## 3. Discussion

There were a number of issues worth noting in the treatment of two patients with anophthalmic enophthalmos. First, eye prosthesis was prone to be customized as thick as possible to camouflage sunken eye. Second, the heavier prosthesis could have induced lower eyelid retraction, worsening the downward displacement of the prosthesis. Third, a prosthetic cornea was redesigned to camouflage the scleral show between the cornea and lower eyelid margin, which could increase the noticeability of hypoglobus, or downgazing appearance. Fourth, mechanical stress arising from attaching and detaching the bulky prosthesis could have led to dehiscence of levator aponeurosis, given that the effect of aponeurotic surgery observed in the first patient was mostly diminished during the first few postoperative months.

 These clinical findings indicate that at least three factors should be taken into consideration in the correction of anophthalmic enophthalmos. (1) Augmentation of the eye socket is the top priority, because restoring adequate orbital volume and convexity of the socket is essential to limit the use of a bulky prosthesis, which can cause various complications. We prefer autologous costal cartilage grafts to alloplastic implants for augmentation because of their low risk for extrusion and less resorption [3, 4]. However, flap transfer may be required depending on the remaining volume of the orbit and the presence of conjunctival contracture [1, 5]. (2) Lower eyelid retraction must be corrected if present, as its importance was demonstrated in our first patient. Although several methods are available to correct lower eyelid retraction [6–8], lower eyelid support with an auricular cartilage is considered advantageous given the long-lasting effect against drooping caused by mechanical stress [7]. (3) Upper eyelid ptosis can be severe. In the case of our first patient, the levator aponeurosis repair had little effect during the first few months. Considering that even daily use of a contact lens can cause aponeurosis dehiscence, there is no doubt that a bulky and irregular prosthesis could induce severe ptosis. Therefore, it should be noted that the effects of aponeurotic surgery may be limited, and thus, frontalis suspension should be indicated more frequently.

 In conclusion, our findings suggest that the following three factors should be considered in the correction of anophthalmic enophthalmos: the remaining orbital volume and socket contracture, the presence of lower eyelid retraction, and severity of upper eyelid ptosis.

## Figures and Tables

**Figure 1:**
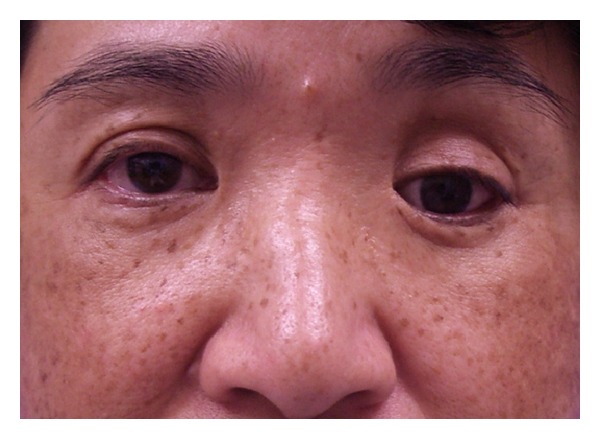
Preoperative view of Case  1.

**Figure 2:**
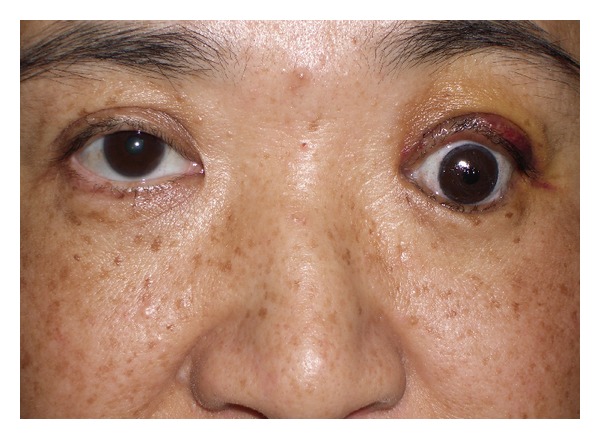
Postoperative 5 days after aponeurotic surgery (Case  1).

**Figure 3:**
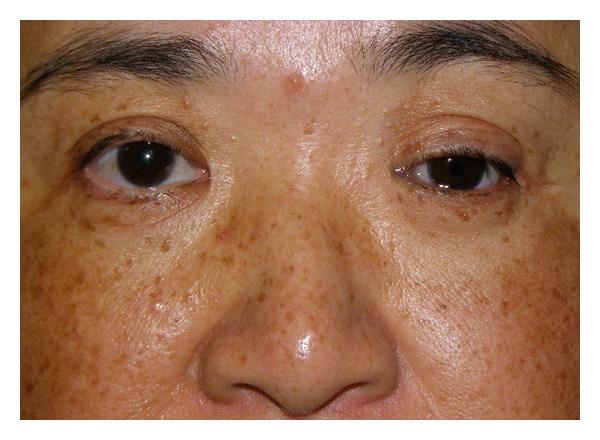
Postoperative 6 months after aponeurotic surgery. Dehiscence of repaired aponeurosis developed (Case  1).

**Figure 4:**
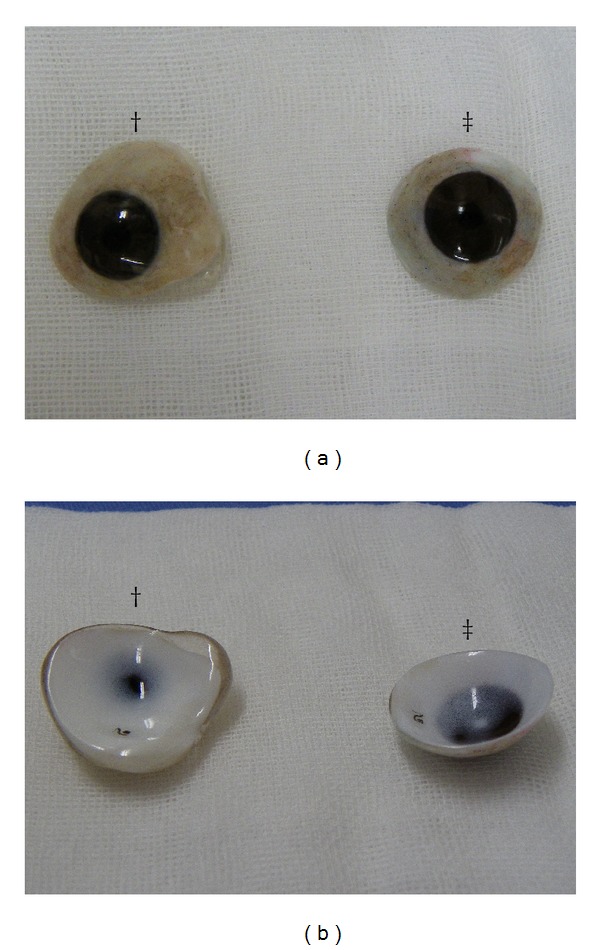
Preoperative (dagger) and final eye prosthesis (double dagger). Note thicker, malformed “sclera,” and nonconcentric “cornea”, preoperatively.

**Figure 5:**
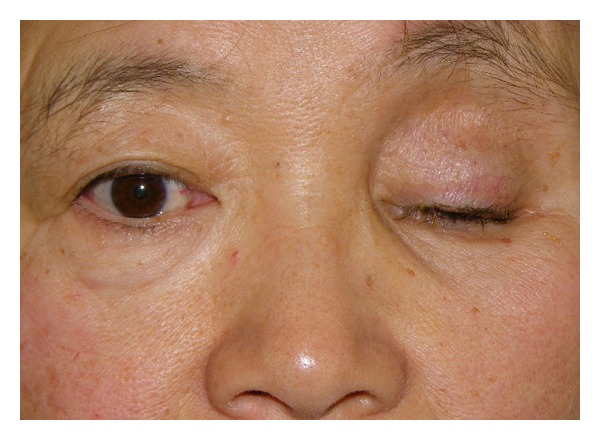
Preoperative view of Case  2.

**Figure 6:**
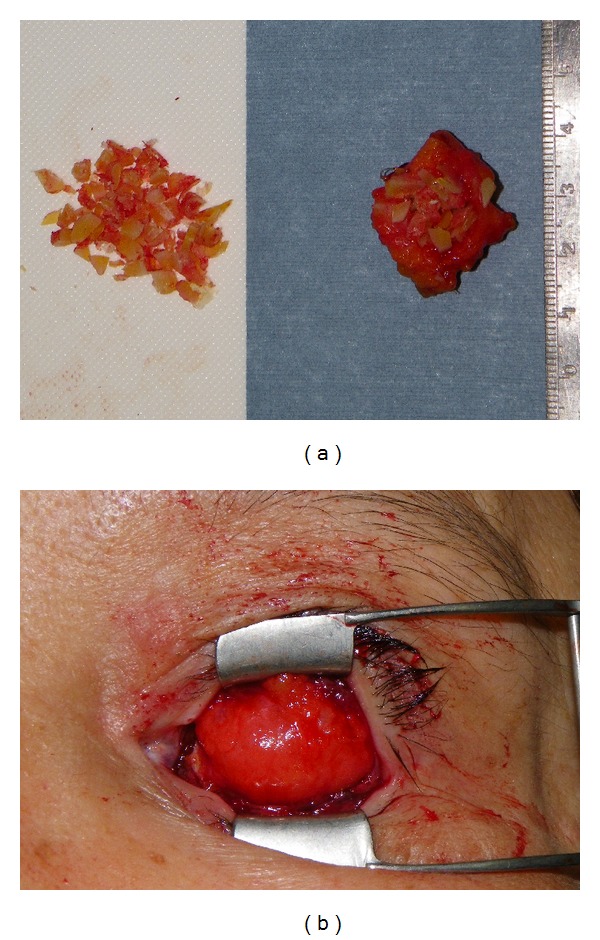
Intraoperative views of the first surgery (Case  2). (a) Diced costal cartilage fragments wrapped with serratus fascia. (b) Prefabricated ball-shaped graft was placed into anophthalmic socket.

**Figure 7:**
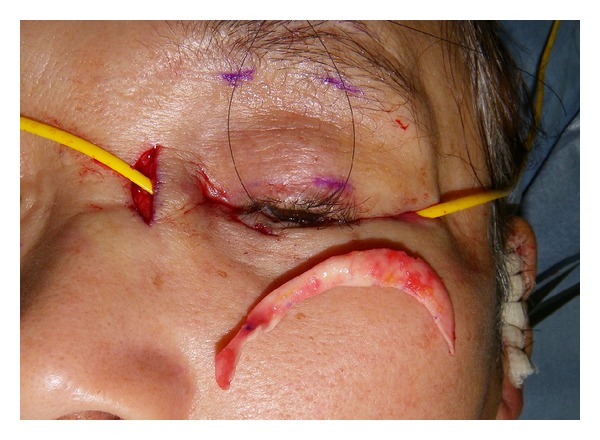
Intraoperative view of the second surgery (Case  2). Lower eyelid support with an auricular cartilage, and frontalis sling for upper eyelid ptosis.

**Figure 8:**
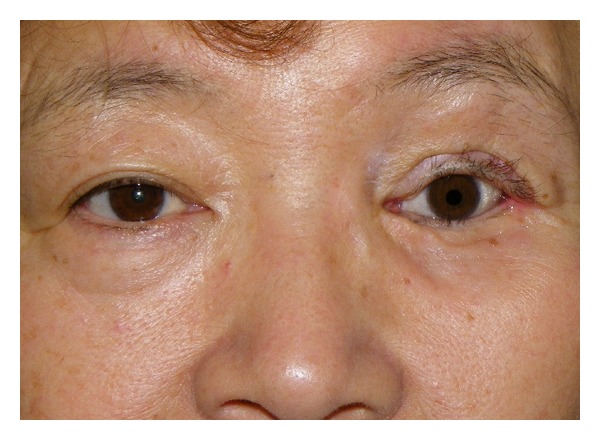
Postoperative view at 17-months followup (Case  2).
